# Composition of Rumen Bacterial Community in Dairy Cows With Different Levels of Somatic Cell Counts

**DOI:** 10.3389/fmicb.2018.03217

**Published:** 2018-12-24

**Authors:** Yifan Zhong, Mingyuan Xue, Jianxin Liu

**Affiliations:** MoE Key Laboratory of Molecular Animal Nutrition, College of Animal Sciences, Institute of Diary Science, Zhejiang University, Hangzhou, China

**Keywords:** somatic cell counts, subclinical mastitis, milk production, rumen fermentation, rumen microbiota

## Abstract

Mastitis is an inflammatory disease, affects the dairy industry and has a severe economic impact. During subclinical mastitis, milk production and milk quality deteriorates. Recently, rumen microbial composition has been linked to rumen health, but few studies have investigated the effect of rumen microbiota on mammary health in cows. This study was undertaken to identify the rumen microbial composition and associated microbial fermentation in cows with different somatic cell counts (SCC), with the speculation that cows with different health statuses of the mammary gland have different rumen bacterial composition and diversity. A total of 319 Holstein dairy cows fed the same diet and under the same management were selected and divided into four groups as SCC1 (*N* = 175), SCC2 (*N* = 49), SCC3 (*N* = 49), and SCC4 (*N* = 46) with < 200,000, 200,001–500,000, 500,001–1,000,000, and >1,000,000 somatic cells/mL, respectively. Further, 20 cows with the lowest SCC and 20 cows with the highest SCC were identified. The rumen microbial composition was profiled using 16S rRNA sequencing, along with measurement of rumen fermentation parameters and milking performance. Compared to low SCC, cows with high SCC showed poorer milk yield, milk composition, and rumen volatile fatty acids concentration, but higher rumen bacterial diversity. Although the predominant rumen bacterial taxa did not vary among the SCC groups, the relative abundance of phyla SR1 and *Actinobacteria*, unclassified family *Clostridiales* and genus *Butyrivibrio* were significantly different. In addition, *Proteobacteria* and family *Succinivibrionaceae* were enriched in cows with low SCC. Our results suggest that specific rumen microbes are altered in cows with high SCC.

## Introduction

Mastitis of dairy cows is an inflammatory disease caused by intramammary infection (IMI), with high incidence and prevalence. It involves a host-pathogen interaction driven by host and bacterial determinants. Subclinical mastitis is characterized by no visible changes in the appearance of milk or the udder, but milk production decreases, milk composition is altered, somatic cell count increases (SCC), and pathogens may be present in the secretion (Batavani et al., 2007). The IMI in dairy cows can cause severe economic losses due to reduced milk production, discarded milk, lower conception rates, premature culling, and treatment costs (Seegers et al., 2003; Halasa et al., 2007). In the dairy industry, SCC have been extensively used to distinguish healthy quarters from quarters with an inflammatory response due to IMI (Tsenkova et al., 2001).

Recently, the fundamental role of naturally occurring diverse gastrointestinal microbes in the well-being of ruminants and humans has been demonstrated (Clemente et al., 2012; Mao et al., 2015). The constituents of microbiota have been shown to interact with each other and with the host immune system in ways that influence the development of disease. In human studies, it has been reported that milk bacteria as well as mastitis can be affected by gut microbiota (Rodriguez, 2014). The oral administration of strains of probiotics isolated from milk has been shown to be an alternative method for the treatment of human mastitis (Arroyo et al., 2010). These studies lead to the speculation that there could be an interaction between gut microbiota and mammary gland, although the mechanism remains under-investigated.

The rumen is considered as one of the major organs in dairy cows, which directly affect milk production and health of the host. Dairy cows depend on rumen microbes to convert human-indigestible feedstock to human-edible products, by providing volatile fatty acids (VFAs) and microbial proteins. In recent years, major efforts in characterizing the rumen microbial composition and function have been made, along with studying the factors that affect rumen microbiota, such as diet (Zhu et al., 2017), lactation stage, rumen development and age (in pre-weaned calves) (Robert et al., 2012). In addition, host-microbial interactions in the rumen have been studied, aiming to maximize the performance of dairy cows, including lactation performance (Xue et al., 2018), feed efficiency, and regulation of methane emission (McCann et al., 2014). Rumen microbial composition has also been linked to the health of rumen (acidosis and subacute acidosis) (Mao et al., 2015). To our knowledge, it is unknown whether the rumen microbiota could be associated with mammary health of the host.

In the current study, it is hypothesized that rumen microbial composition, diversity, and rumen fermentation parameters differed in cows with different health statuses of mammary gland (different levels of SCC). The16S rRNA gene sequencing was performed to investigate the rumen bacterial composition, and microbial fermentation products were characterized in 319 dairy cows with different levels of SCC. The current study provides a better understanding of rumen microbial composition and fermentation in cows with different health statuses of mammary gland.

## Materials and Methods

### Ethics Statement

The experimental procedures used in this study were approved by the Animal Care Committee of Zhejiang University (Hangzhou, China) and were in accordance with the University's guidelines for animal research.

### Experimental Design

A total of 319 multiparous (parity ranged from 2 to 7) Holstein dairy cows in mid-lactation (day in milk [DIM] ranged from 92 to 218) with no clinical signs of disease were selected from 652 lactating cows in a commercial dairy farm (Hangzhou, China). The animals were fed the same diet three times daily at 0630, 1400 and 2000 h, with forage to concentrate ratio of 45:55 (Supplementary Table S1), and had free access to water. Animals were divided into four groups according to the SCC in milk, including 175 cows with SCC ranging from 0 to 200,000 cells/mL (SCC1), 49 cows with SCC ranging from 200,001 to 500,000 cells/mL (SCC2), 49 cows with SCC ranging from 500,001 to 1,000,000 cells/mL (SCC3), and 46 cows with SCC greater than 1,000,000 cells/mL (SCC4). The SCC cut-offs were established based on a previous study on SCC values in healthy quarters (Hand et al., 2012). In order to increase the statistical power when comparing the rumen microbiota and fermentation (Supplementary Table S2), cows with extremely low and high SCC (including 20 cows with the lowest SCC [L_SCC] and 20 cows with the highest SCC [H_SCC]) were selected from the 319 cows based on the extreme phenotype characterization approach (Shabat et al., 2016). A previous study has reported that rumen samples from 16 cows are sufficient to cover the microbial diversity in rumen fed the same diet (Jami and Mizrahi, 2012).

### Sample Collection and Analysis

Milk yield of all the 319 cows was recorded for three consecutive days. Milk samples of all the 319 cows were collected on the third day (sampling day) for the measurement of milk protein, fat, lactose, urea nitrogen (MUN) and SCC by infrared analysis (Laporte and Paquin, 1999) using a spectrophotometer (Foss-400; Foss Electric A/S, Hillerod, Denmark).

Rumen content of all the 319 cows was collected using oral stomach tubes (Shen et al., 2012) before the morning feeding on the sampling day. The pH of the rumen fluid was measured immediately using a pH meter (FE20-FiveEasy Plus™; Mettler Toledo Instruments Co. Ltd., Shanghai, China). The rumen content of all the 319 cows were used for analysis of VFAs and ammonia nitrogen (NH_3_-N). One set of triplicate samples of 1 mL were acidified with 20 μL orthophosphoric acid for analysis of VFAs, one set of triplicate samples of 1 mL were acidified with 6 N HCl and frozen at −20°C for analysis of NH_3_-N, as described by Hu et al. (2005). The third set of triplicate samples of 1 mL were frozen at −80°C until DNA extraction.

### DNA Extraction and Sequencing

Total DNA was extracted from rumen content of all the 319 cows using the bead-beating method (Li et al., 2009). DNA qualities and quantities were measured using the NanoDrop 2000 Spectrophotometer (NanoDrop Technologies, Wilmington, DE, USA). The DNA was amplified using the 341F/806R primer set (341 F: 5′-CCTATYGGGRBGCASCAG-3′, 806R: 5′-GGACTACNNGGGTATCTAAT-3′), which targets the V3-V4 region of the bacterial 16S rRNA gene. The reaction contained 0.5 U of Taq polymerase (TransGen Biotech, Beijing, China) in 25 μL of 10 × PCR buffer, 200 μM of each dNTP, 0.2 μM of each primer and 2 μL of DNA (50 ng/μL). Double distilled water was added to make the volume reach 25 μL. The PCR reactions were performed using Phusion High-Fidelity PCR MasterMix (New England Biolabs LTD., Beijing, China) with the following program, 94°C for 3 min, 35 cycles of 94°C for 45 s, 50°C for 60s and 72°C for 90s, followed by 72°C for 10 min. The PCR products were visualized on 2% agarose gels and purified using the QIAquick Gel Extraction Kit (QIAGEN, Dusseldorf, Germany). Amplicon sequencing was conducted on an Illumina HiSeq platform using the paired-end 2 × 250 bp sequencing (Caporaso et al., 2012).

### Analysis of Sequencing Data

Paired-end reads were merged using FLASH (V1.2.7, http://ccb.jhu.edu/software/FLASH/) (Magoč and Salzberg, 2011). Sequences were de-multiplexed and quality-filtered using QIIME (version 2, http://qiime.org/index.html) and bases with quality scores higher than 20 were retained for further analysis (Caporaso et al., 2010). Chimeric sequences were identified and removed using the UCHIME algorithm (UCHIME Algorithm, http://www.drive5.com/usearch/manual/uchime_algo.html) (Edgar et al., 2011). Operational taxonomic units (OTUs) were clustered based on 97% similarity threshold using UPARSE (Uparse v7.0.1001, http://drive5.com/uparse/) (Edgar, 2013), and taxonomy was assigned using the latest Greengenes database (http://greengenes.secondgenome.com Greengenes May 2013 release). The OTU-level alpha diversity of the bacterial communities was determined using various diversity indices (Chao1, Shannon, Simpson and Ace indexes) and calculated using procedures within QIIME (version 2). Jack-knifed beta diversity was calculated based on OTU-level weighted and unweighted Unifrac distances using QIIME (version 2), and visualized by principal coordinates analysis (PCoA) using PAST software (version 3.18, http://folk.uio.no/ohammer/past).

### Statistical Analysis

Lactation performance (DIM, parity, milk yield, milk fat, milk protein, lactose, MUN and SCC), total and individual rumen VFA (acetate, propionate, butyrate, isobutyrate, valerate, and isovalerate) concentrations and alpha diversity indices of the four SCC groups were analyzed using one-way ANOVA; and the relative abundances of bacterial taxa among the four SCC groups were compared using Kruskal-Wallis H test (four SCC groups). Scheffe's method was used for multiple comparisons of means among the four SCC groups. Lactation performance, total and individual rumen VFAs concentrations and alpha diversity indices in H_SCC and L_SCC groups were analyzed using t-test; and the relative abundances of bacterial taxa between the two groups were compared using Wilcoxon rank-sum test. Statistical analysis was performed using SPSS (version 22), with statistical significances declared at *P* < 0.05. The *P*-values from the Kruskal-Wallis H test and Wilcoxon rank-sum test were adjusted by the false discovery rate (FDR < 0.05) (Benjamini and Hochberg, 1995), with statistical significances declared at FDR-adjusted *P* < 0.05. Linear discriminant analysis effect size (LEfSe) was used to further compare relative abundances of microbial taxa in H_SCC and L_SCC groups, and significant differences were considered by a linear discriminant analysis (LDA) score > 2 and *P* < 0.05.

## Results

### Measurement of SCC, Lactation Performance and Rumen VFAs

The parities of the 319 dairy cows were 3.1 ± 0.07 (mean ± standard error of the mean [SEM]), and the DIMs of the 319 dairy cows were 160 ± 1.87 (Table 1). Milk yield in SCC1 was the highest among the four groups, followed by SCC2 and SCC3, and the lowest in SCC4 (*P* < 0.01). Milk fat, milk protein and MUN content were significantly higher (*P* < 0.01), and the lactose content was significantly lower (*P* < 0.01) in SCC4 compared to the other 3 groups (Table 1). Rumen pH, and concentration of VFAs and NH_3_-N were not different among the four SCC groups. With the increase in SCC, the acetate to propionate (A: P) ratio increased, with higher A: P ratio in SCC4 (*P* < 0.05) than in SCC1 (Table 1).

**Table 1:** The somatic cell counts (SCC), milk yield, milk composition, and rumen fermentation parameters of the lactating dairy cows.

**Items^**1**^**	**Groups**^****2****^				**SEM**	***P*-value**	**Contrasts**		**Groups**^****3****^		**SEM**	***P*-value**
	**SCC1**	**SCC2**	**SCC3**	**SCC4**			**L**	**Q**	**L_SCC**	**H_SCC**		
	***n* = 175**	***n* = 49**	***n* = 49**	***n* = 46**					***n* = 20**	***n* = 20**		
SCC (× 10^3^/mL)	71.5^d^	332.5^c^	704.8^b^	3107.6^a^	78.9	< 0.01	< 0.01	< 0.01	17.7^b^	5021.1^a^	503.8	< 0.01
Parity	2.84^b^	3.41^a^	3.55^a^	3.35^a^	0.07	< 0.01	0.01	0.01	2.80	3.40	0.15	0.10
DIM	154.7^b^	160.1^a^^b^	165.1^a^^b^	171.0^a^	1.87	< 0.01	< 0.01	0.96	163.7	162.2	5.03	0.88
Milk yield (kg/d)	34.5^a^	30.1^b^	30.2^b^	25.3^c^	0.45	< 0.01	< 0.01	0.81	38.8^a^	23.6^b^	1.96	< 0.01
**MILK COMPOSITION (%)**												
Milk fat	3.07^b^	3.21^b^	3.09^b^	3.73^a^	0.05	< 0.01	< 0.01	0.03	3.01^b^	3.70^a^	0.14	0.01
Milk protein	3.07^b^	3.13^b^	3.11^b^	3.32^a^	0.02	< 0.01	< 0.01	0.04	3.04^b^	3.38^a^	0.05	< 0.01
Lactose	5.04^a^	4.98^b^	4.92^b^	4.77^c^	0.01	< 0.01	< 0.01	0.06	5.06^a^	4.65^b^	0.06	< 0.01
MUN^3^ (mg/dL)	14.5^a^	14.1^a^^b^	14.0^a^^b^	13.5^b^	0.12	0.04	0.01	0.81	14.9^b^	13.4^b^	0.41	0.03
Rumen pH	6.87	6.88	6.89	6.90	0.01	0.89	0.89	0.89	6.90	6.93	0.03	0.70
**VFA CONCENTRATION (MMOL/L)**												
Acetate	70.5	72.3	70.7	71.0	0.61	0.79	0.98	0.61	69.2	67.9	1.42	0.66
Propionate	17.4	18.1	17.2	16.2	0.25	0.29	0.06	0.17	17.7^a^	15.1^b^	0.63	0.03
Isobutyrate	0.96	0.93	0.96	0.99	0.01	0.69	0.38	0.32	0.96	0.98	0.05	0.82
Butyrate	11.0	10.8	10.6	11.1	0.14	0.67	0.99	0.27	10.7	10.3	0.32	0.56
Isovalerate	1.46	1.49	1.48	1.47	0.02	0.92	0.90	0.60	1.43	1.39	0.03	0.61
Valerate	1.16	1.20	1.13	1.10	0.01	0.15	0.04	0.29	1.17^a^	1.03^b^	0.03	0.04
Total VFA	102.8	105.0	100.2	101.9	1.04	0.62	0.45	0.92	101.1	96.7	2.21	0.32
A: P Ratio	4.11^b^	4.16^a^^b^	4.29^a^^b^	4.49^a^	0.04	< 0.01	< 0.01	0.43	4.05^b^	4.58^a^	0.11	0.02
Ammonia-N (mg/dL)	6.67	6.76	6.81	6.71	0.11	0.96	0.86	0.68	6.54	6.38	0.22	0.73

*^1^VFA, volatile fatty acid; MUN, milk urea nitrogen; NH_3_-N, ammonium nitrogen. ^2^SCC1, somatic cell counts < 200,000/mL; SCC2, somatic cell counts range from 200,001 to 500,000/mL; SCC3, somatic cell counts range from 500,001 to 1,000,000/mL; SCC4, somatic cell counts >1,000,000/mL. ^3^L_SCC, lowest SCC; H_SCC, highest SCC*.

a−c *Means with different superscript letter differ (P < 0.05) within groups*.

Milk yield (*P* < 0.01), lactose content (*P* < 0.01) and MUN (*P* = 0.03) were significantly higher in L_SCC, whereas the content of milk fat (*P* = 0.01) and milk protein (*P* < 0.01) were significantly higher in H_SCC. Molar proportion of propionate (*P* = 0.03) and valerate (*P* = 0.04) were significantly higher in L_SCC group than in H_SCC, whereas the A: P ratio was significantly higher in H_SCC (*P* = 0.02, Table 1).

### Rumen Bacterial Communities in Cows With Different Levels of SCC

The amplicon sequencing of rumen samples generated a total of 19,253,662 high-quality sequences across all samples, and an average of 1,826 OTUs per sample, with a Good's coverage of 99.9% across all samples. A total of 26 bacterial phyla were identified across all the samples, with *Firmicutes* (49.8 ± 0.45%) and *Bacteroidetes* (33.6 ± 0.48%) being the most abundant and *Proteobacteria* (9.50 ± 0.38%), *Tenericutes* (2.16 ± 0.04%) and *Spirochaetes* (1.42 ± 0.04%) being less abundant (Table 2). At genus level, a total of 415 genera were identified. The bacterial genera with relative abundance > 1% were considered as predominant, accounting for 85.9 ± 0.13% of the total sequences. Among the predominant genera, *Prevotella* (19.3 ± 0.48%), unclassified_o_*Clostridiales* (9.16 ± 0.11%), *Ruminococcus* (8.37 ± 0.10%), unclassified_f_*Succinivibrionaceae* (8.03 ± 0.38%), and unclassified_f_*Ruminococcaceae* (7.95 ± 0.12%) were the most abundant.

**Table 2:** Phylum and genus composition (Relative abundances >1%) of the rumen bacteria in cows with different somatic cell counts (SCC).

**Items**	**Groups**^****1****^ **(%)**				**SEM**	***P*-value**	**Groups**^****2****^ **(%)**		**SEM**	***P*-value**
	**SCC1**	**SCC2**	**SCC3**	**SCC4**			**L_SCC**	**H_SCC**		
	***n* = 175**	***n* = 49**	***n* = 49**	***n* = 49**			***n* = 20**	***n* = 20**		
***Firmicutes***	49.9	48.8	49.3	50.9	0.45	0.60	48.2	49.7	1.21	0.53
unclassified_o__*Clostridiales*	9.27	9.03	8.96	9.11	0.11	0.72	8.92	8.65	0.29	0.67
*Ruminococcus*	8.39	8.23	8.17	8.67	0.10	0.55	7.78	8.40	0.26	0.25
unclassified_f__*Ruminococcaceae*	7.86	7.62	8.01	8.57	0.11	0.12	7.37	8.40	0.38	0.18
unclassified_f__*Lachnospiraceae*	3.29	3.32	3.39	3.49	0.03	0.15	3.24	3.38	0.08	0.39
unclassified_o__*Clostridiales*	3.06	2.86	2.95	3.23	0.05	0.13	2.93	3.28	0.14	0.22
*Butyrivibrio*	3.05^a^	2.85^a^^b^	2.71^b^	3.05^a^	0.04	0.03	2.93	3.08	0.12	0.56
*Coprococcus*	2.24	2.25	2.39	2.23	0.03	0.27	2.31	2.13	0.07	0.17
unclassified_f__*Lachnospiraceae*	2.09	2.09	2.10	2.08	0.02	0.99	2.08	1.99	0.05	0.37
*Clostridium*	1.33	1.32	1.39	1.39	0.02	0.39	1.28	1.37	0.05	0.46
unclassified_f__*Ruminococcaceae*	1.26	1.23	1.28	1.34	0.02	0.29	1.20	1.36	0.06	0.16
*Shuttleworthia*	1.23	1.35	1.26	1.01	0.01	0.47	1.43	1.02	0.15	0.18
unclassified_f__[*Mogibacteriaceae*]	0.95	0.92	1.01	1.01	0.01	0.06	0.88	1.00	0.04	0.14
***Bacteroidetes***	33.3	33.4	33.7	34.6	0.49	0.86	32.0	36.5	1.47	0.13
*Prevotella*	19.2	19.4	18.7	19.9	0.48	0.94	19.0	21.5	1.35	0.35
unclassified_o__*Bacteroidales*	5.64	5.70	5.94	5.66	0.08	0.66	5.20	5.62	0.19	0.29
unclassified_o__*Bacteroidales*	2.49	2.49	2.79	2.77	0.05	0.10	2.23^b^	2.84^a^	0.16	0.05
unclassified_f__S24-7	1.39	1.18	1.32	1.38	0.03	0.06	1.29	1.52	0.09	0.19
CF231	1.00	1.03	1.07	1.08	0.02	0.61	0.98	1.02	0.07	0.67
unclassified_f__RF16	0.97	0.99	1.08	1.08	0.02	0.10	0.91^b^	1.13^a^	0.05	0.03
***Proteobacteria***	9.70	10.6	9.77	7.30	0.38	0.10	12.95^a^	6.85^b^	1.34	0.02
unclassified_f__*Succinivibrionaceae*	8.27	9.04	8.26	5.80	0.00	0.09	11.49^a^	5.47^b^	1.34	0.02
***Tenericutes***	2.13	2.24	2.18	2.15	0.04	0.84	2.06	2.02	0.13	0.89
unclassified_o__RF39	1.55	1.63	1.62	1.59	0.04	0.86	1.53	1.48	0.12	0.89
***Spirochaetes***	1.39	1.48	1.42	1.41	0.04	0.93	1.44	1.28	0.12	0.56
*Treponema*	1.38	1.46	1.41	1.39	0.04	0.95	1.42	1.26	0.12	0.48

*^1^SCC1, somatic cell counts < 200,000/mL; SCC2, somatic cell counts range from 200,001 to 500,000/mL; SCC3, somatic cell counts range from 500,001 to 1,000,000/mL; SCC4, somatic cell counts >1,000,000/mL. ^2^L_SCC, lowest SCC; H_SCC, highest SCC*.

a−c *Means with different superscript letter differ (P < 0.05) within groups*.

**Figure 1:**
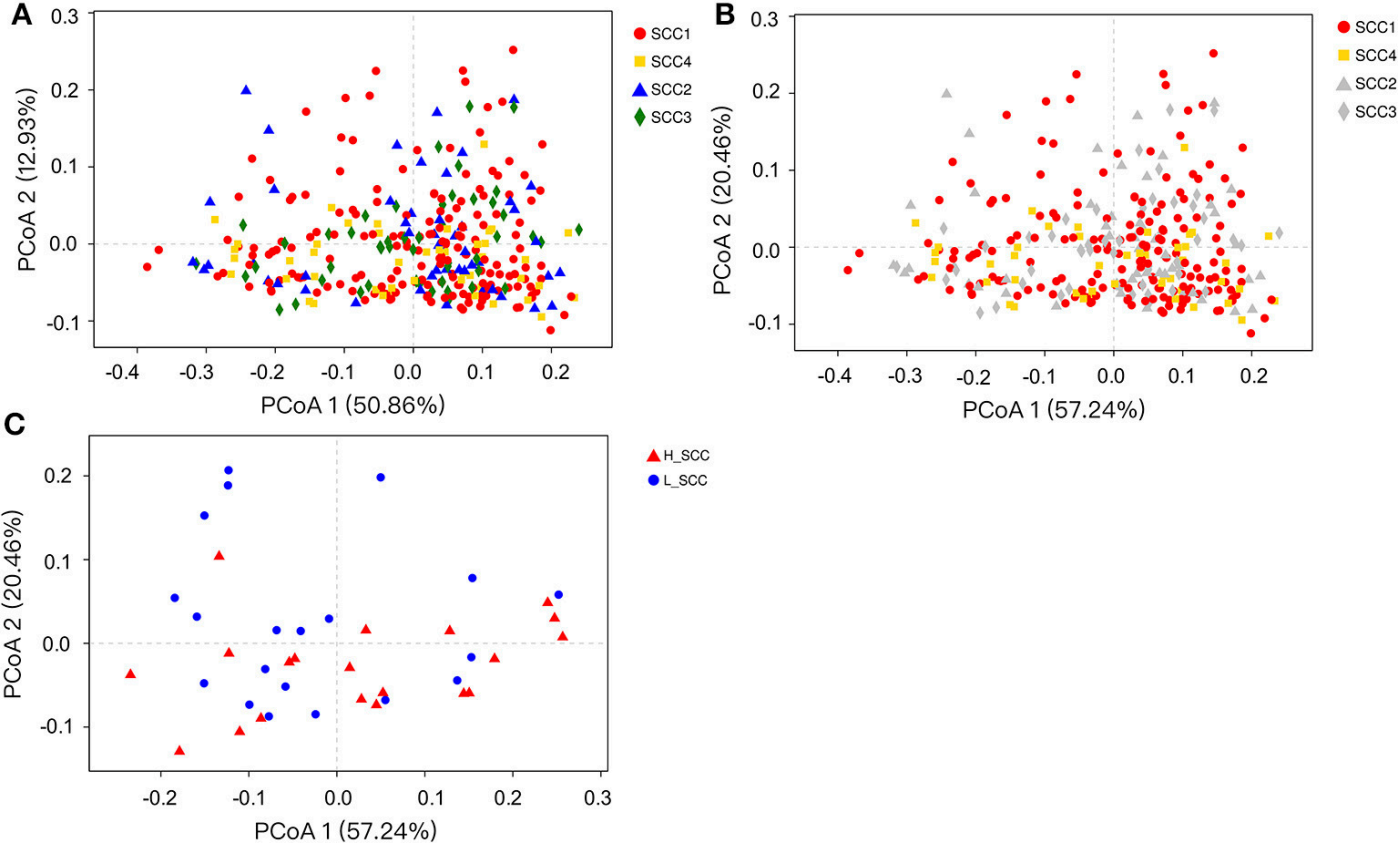
Principal coordinates analysis (PCoA) based on weighted Unifrac distances of OTUs. Samples are indicated by points and colored for different SCC groups. **(A)** PCoA of the four SCC groups. **(B)** PCoA of the four SCC groups with SCC1 and SCC4 highlighted. **(C)** PCoA of L_SCC and H_ SCC groups.

The comparison of alpha diversity indices showed a significant difference of Shannon index among the four SCC groups at OTU level (Table 3). The Shannon index was significantly higher in SCC4 (P = 0.04) compared to SCC1. The PCoA based on weighted (Figure 1A) and unweighted Unifrac distances (Supplementary Figure S1A) showed no clear separation among the four SCC groups or between SCC1 and SCC4 (Figure 1B and Supplementary Figure S1B). Specific bacterial genera were unique to the rumen of cows with different levels of SCC (Supplementary Table S3), and included 29, 6, 10 and 8 unique genera in SCC1, SCC2, SCC3 and SCC4 groups, respectively. However, these unique bacterial genera in the different SCC groups were in low abundance, with relative abundance < 0.0002%.

**Table 3:** Bacterial alpha diversity of the cows with different somatic cell counts (SCC).

**Items**	**Groups**^****1****^				**SEM**	***P*-value**	**Contrasts**		**Groups**^****2****^		**SEM**	***P*-value**
	**SCC1**	**SCC2**	**SCC3**	**SCC4**			**L**	**Q**	**L_SCC**	**H_SCC**		
	***n* = 175**	***n* = 49**	***n* = 49**	***n* = 46**					***n* = 20**	***n* = 20**		
Shannon	6.19^b^	6.18^b^	6.25^ab^	6.31^a^	0.02	0.04	< 0.01	0.35	6.07^b^	6.31^a^	0.06	0.03
Simpson	0.009	0.010	0.008	0.006	0.00	0.14	0.02	0.43	0.015^a^	0.007^b^	0.00	0.04
Ace	2,528	2,530	2,572	2,563	6.61	0.06	0.02	0.71	2555	2596	17.0	0.26
Chao 1	2,582	2,573	2,627	2,620	7.25	0.07	0.02	0.95	2612	2655	19.6	0.27

*^1^SCC1, somatic cell counts < 200,000/mL; SCC2, somatic cell counts range from 200,001 to 500,000/mL; SCC3, somatic cell counts range from 500,001 to 1,000,000/mL; SCC4, somatic cell counts >1,000,000/mL. ^2^L_SCC, lowest SCC; H_SCC, highest SCC*.

a-c *Means with different superscript letter differ (P < 0.05) within groups*.

The Shannon index was significantly higher (P = 0.03), and the Simpson index significantly lower in H_SCC (*P* = 0.04) compared to the L_SCC group (Table 3). The PCoA based weighted (Figure 1C) and unweighted Unifrac distances (Supplementary Figure S1C) showed no clear separation between L_SCC and H_SCC groups. In addition, 64 and 31 unique bacterial genera existed in L_SCC and H_SCC groups, respectively (Supplementary Table S4).

### Differential Rumen Bacterial Taxa in Cows With Different Levels of SCC

**Figure 2:**
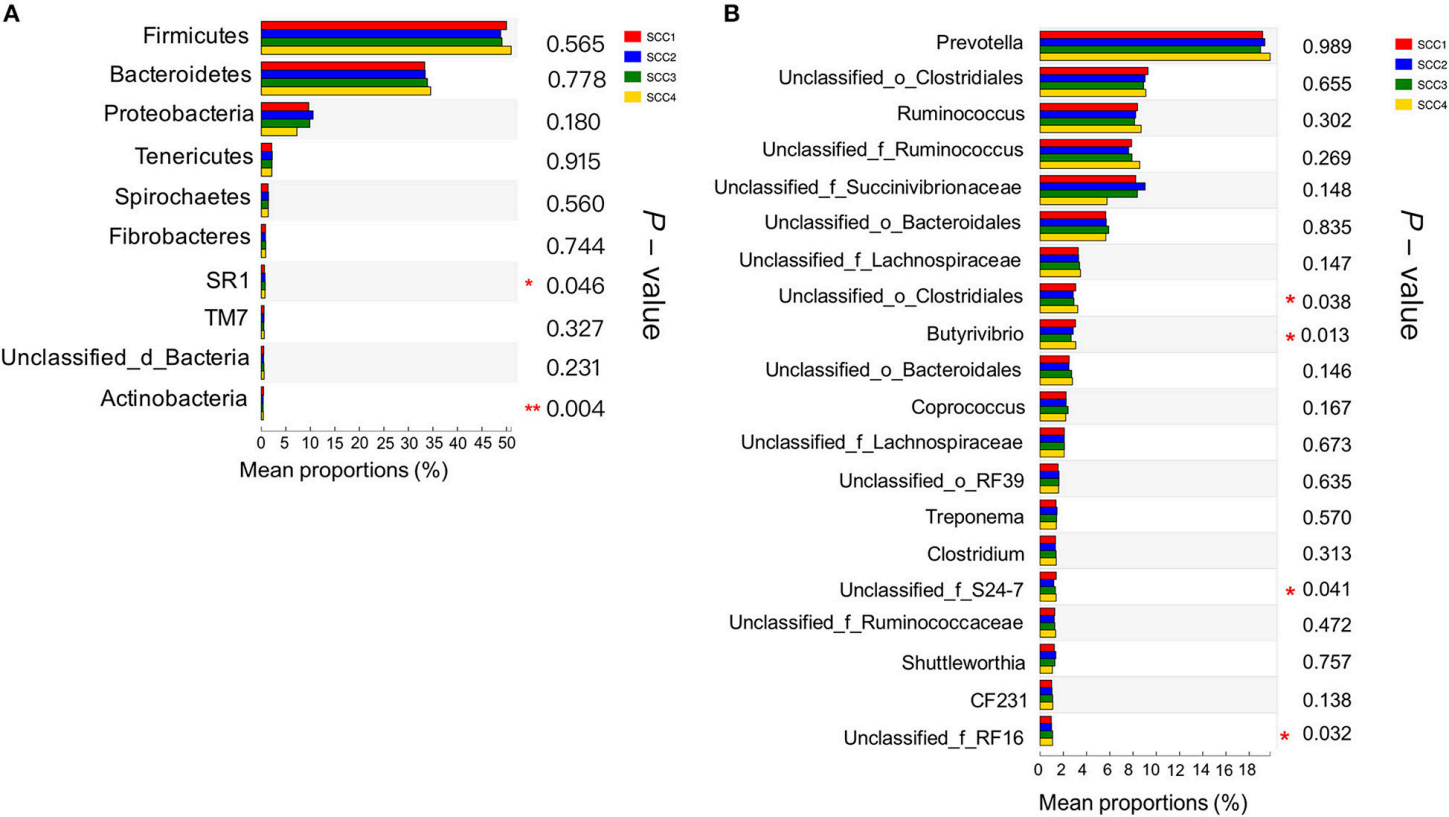
Comparison of relative abundances of bacterial phyla and genera in the different SCC groups. **(A)** The 10 most abundant phyla in the four groups. **(B)** The 20 most abundant genera are presented. *0.01 < *P* ≤ 0.05, **0.001 < *P* ≤ 0.01.

Further, Kruskal-Wallis H analysis of the relative abundances of phyla revealed that phyla SR1 (FDR-adjusted *P* = 0.05) and *Actinobacteria* (FDR-adjusted *P* < 0.01) were significantly different among the four SCC groups (Figure 2A). At genus level, *Butyrivibrio* was significantly higher in SCC4 (FDR-adjusted *P* = 0.02, Figure 2B). Three unclassified taxa belonging to the families *Clostridiales* (FDR-adjusted *P* = 0.04), S24-7 (FDR-adjusted *P* = 0.04) and RF 16 (FDR-adjusted *P* = 0.03) were significantly different among the four SCC groups.

**Figure 3:**
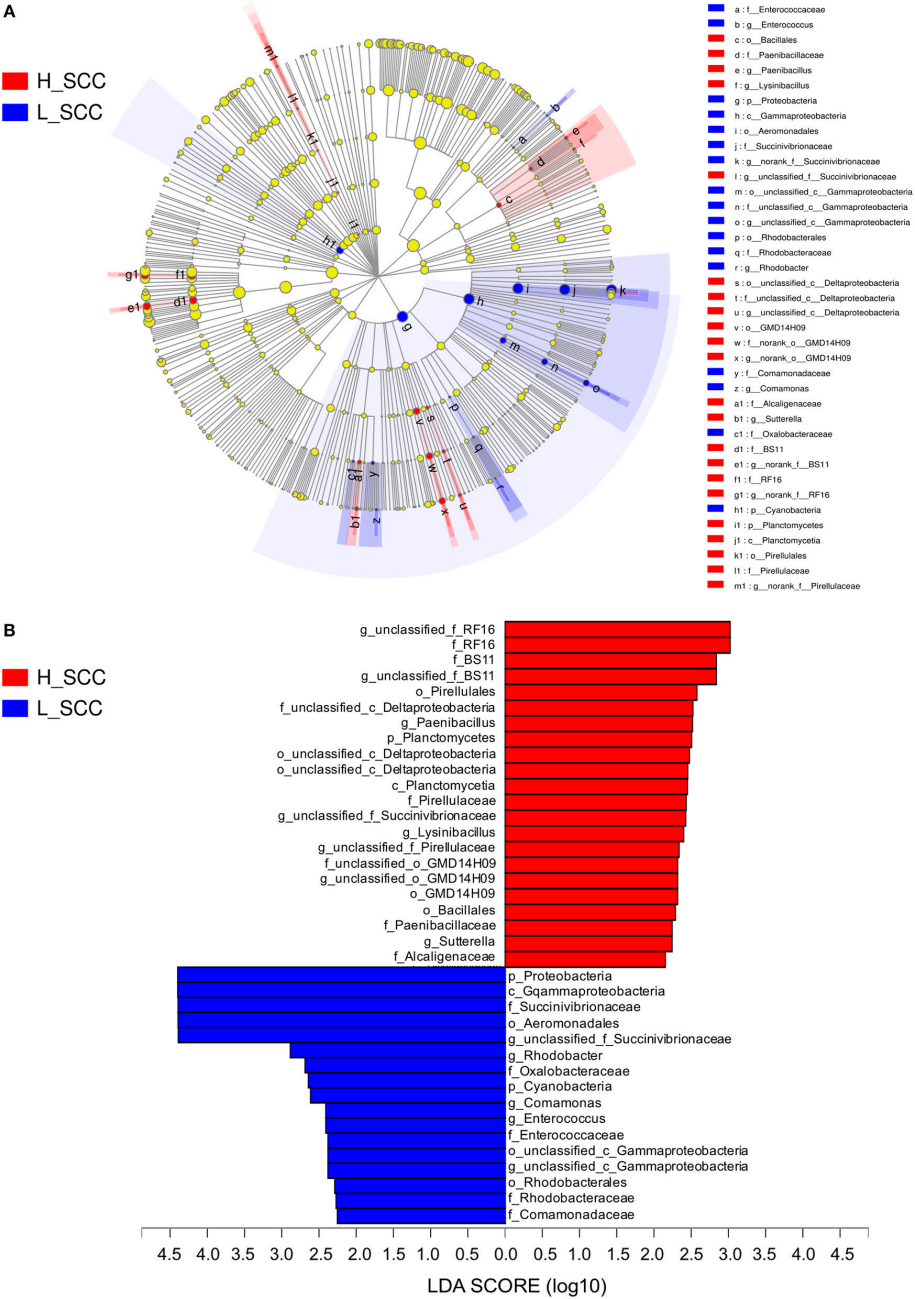
The LDA effect size (LEfSe) analysis of bacterial taxa between L_SCC and H_SCC groups. **(A)** Cladogram shows significantly enriched bacterial taxa (from phylum to genus level). **(B)** Bar chart shows LDA score of L_SCC and H_SCC groups. Significant differences are defined as *P* < 0.05 and LDA score > 2.0.

The most differentially abundant bacterial taxa in the H_SCC group tested by the LEfSe analysis belonged to the genera unclassified_f_RF16, *Paenibacillus*, unclassified_c_*Deltaproteobacteria*, unclassified_f _BS11, unclassified_f _*Succinivibrionaceae, Lysinibacillus*, unclassified_f _*Pirellulaceae*, unclassified_o _GMD14H09 and *Sutterella* (Figure 3). The genera unclassified_f _*Succinivibrionaceae, Rhodobacter, Comamonas, Enterococcus*, and unclassified_c _*Gammaproteobacteria* were more abundant in the L_SCC group (Figure 3). The genera unclassified_f_RF16 and unclassified_f_*Succinivibrionaceae* weighted most to the differences between the communities, with an absolute LDA score factor of approximately 3.

## Discussion

Animal IMI is the major factor that influences SCC (González-Rodríguez et al., 1995), with high SCC (> 200,000 cells/mL) in dairy cows indicating infection and low milk quality (Pyorala, 2003). The SCC at the cow level is commonly used as a tool to diagnose subclinical mastitis in dairy farms. Although the variation of SCC may exist among mammary quarters within a cow, the SCC at cow level can also reflect the general statuses of mammary quarters (Schukken et al., 2003) and is easier to detect relatively compared with SCC at quarter level. In this study, none of the cows in the cohort showed clinical signs of disease, but the cohort had varied SCC (ranging from 8,000 to 11,963,000 cells/mL), indicating that higher SCC cows (> 200,000 cells/mL, including cows in SCC2, SCC3, and SCC4) are at risk of IMI and suffer from subclinical mastitis.

It has been reported that milk yield and composition were negatively affected by IMI, and the degree of changes depended on the inflammatory response (Pyorala, 2003). This change can be the result of damage to mammary epithelial cells by either mastitis-causing bacteria or by the cow's immune response to infection (Gray et al., 2005). It has also been reported that the ruminal VFAs derived from microbial fermentation are also key factors that directly affect the milk biosynthesis (Flint and Bayer, 2008). The VFA concentrations were significantly different between cows with the lowest SCC (L_SCC group) and highest SCC (H_SCC group), with higher concentrations of propionate and valerate, and a lower A: P ratio in the cows of L_SCC group. Such differences in rumen microbial metabolites suggests an altered trend in rumen microbial fermentation in animals at risk of IMI. As the major microbial fermentation product, propionate is the main glucogenic nutrient in ruminants (Danfær, 1994; Reynolds et al., 2003), and glucose is the main precursor for lactose synthesis. The increase in post-hepatic glucose availability could be a potential regulator of milk yield. In this study, the higher rumen propionate, lactose, and better milk yield in cows with lower SCC compared to cows with higher SCC, suggests that IMI cows have a different rumen microbial fermentation pattern from healthy cows, and the fermentation products affect milk synthesis in the mammary gland. These observations could be associated with differential microbial composition and function in the rumen.

When the alpha diversity indices of rumen microbial community were compared, the Shannon index was significantly higher in H_SCC group, and the Simpson index were significantly lower in the L_SCC group. The differences in the alpha diversity indices between the two groups suggested that the rumen microbial population had higher diversity and less strength of dominance in cows with IMI (Rocchini et al., 2015). Additionally, the comparison of rumen bacterial taxa indicated that the predominant phyla (*Firmicutes* and *Bacteroidetes*) along with the well-studied genera were not different in cows with different SCC levels. For example, the main cellulolytic and hemicellulolytic bacteria (*Fibrobacter, Ruminococcus, Clostridium* and *Eubacterium*), amylolytic bacteria (*Streptococcus* and *Ruminobacter*), proteolytic bacteria (*Treponema* and *Lachnospira*) and saccharolytic bacteria (*Succinivibrio, Selenomonas, Lactobacillus*, and *Bifidobacterium*) did not vary among cows with different SCC levels. These results suggest that the bacterial communities, which play a fundamental role in rumen functions, are relatively stable in cows with different health statuses of mammary gland.

Specific microbes, notably at phylum level were observed to be variable among cows with different levels of SCC (four groups). The SCC1 group had lower abundance of SR1 and higher abundance of *Actinobacteria* than the other groups. The phylum SR1 exists in the rumen of many herbivores (Davis et al., 2009), and *Actinobacteria* are regular, though infrequent, members of the rumen microflora, representing up to 3% of total rumen bacteria (Singh et al., 2012). However, information is limited on the ecology and biology of these two taxa (Sulak et al., 2012; Campbell et al., 2013). Further studies to identify the functions of taxa within SR1 and *Actinobacteria* in the rumen are required to determine whether these two taxa are linked to IMI in dairy cows. Additionally, when the H_SCC and L_SCC groups were compared, *Proteobacteria* was significantly enriched in the L_SCC group, and unclassified *Succinivibrionaceae*, the most abundant genera within *Proteobacteria*, was the major contributor to this difference. Microbes belonging to *Succinivibrionaceae* commonly produce succinate, the precursor of propionate (Pope et al., 2011). Although succinate was not measured in this study, propionate was significantly higher in the L_SCC group, suggesting that this taxon could contribute to the higher propionate in the L_SCC group.

Traditionally, the mammary gland is considered a sterile environment, and bacterial cells in milk are considered to be the result of environmental contamination. However, the detection of live bacterial cells and/or DNA from anaerobic species that are usually related to the gut environment in milk has fueled scientific debate on the origin of milk-associated bacteria (Jost et al., 2014). Young et al. (2015) investigated the microbial composition of milk, blood, and feces of healthy lactating cows, and found that OTUs belonging to *Ruminococcus, Bifidobacterium*, and *Peptostreptococcaceae* were present in all three samples from the same animal, suggesting the existence of endogenous entero-mammary pathways during lactation in dairy cows. In a recent study comparing structural and functional features of the gut microbiome between mastitic and healthy cows, Ma et al. (2016) reported that the changes of fecal microbial community of mastitis cows was similar to that of the milk, characterized by a general increase in the mastitis pathogens (*Enterococcus, Streptococcus*, and *Staphylococcus*) and deprivation of *Lactobacillus* and its members. The above studies indicated that the gut microbiota could be associated with different health statuses of mammary gland. Since the gastrointestinal cross-talk in ruminants (including different segments, such as rumen, small and large intestine), along with the relationship between gastrointestinal microbiota and health statuses of mammary gland in ruminants has not been studied in detail, it is therefore recommended that both gut and rumen microbiota are taken into consideration and to investigate the relationship between mammary gland health and gastrointestinal microbiota in further studies.

Although the cows with the lowest and highest SCC in the cohort were selected and compared to get adequate power to detect the rumen microbial components, the power analysis based on the microbiome outcomes still indicated a low power (Supplementary Table S2). Due to the animals in this study showed no clinical signs of mastitis, it is speculated that the rumen microbial community may differ more evidently between healthy cows and cows with clinical mastitis. Such speculations need to be confirmed by further studies comparing rumen microbiota between healthy cows and cows with clinical mastitis.

## Conclusion

Compared to cows with lower SCC (71,460 ± 3,890 cells/mL), cows with higher SCC (3,107,610 ± 368,100 cells/mL) had lower milk yield, poorer milk composition, and reduced rumen VFA concentrations, but higher rumen bacterial diversity. Although the beta diversity and the predominant rumen bacterial taxa did not vary among cows with different SCC levels, the relative abundance of phyla SR1 and *Actinobacteria*, unclassified family *Clostridiales* and genus *Butyrivibrio* were significantly different among the SCC groups. *Proteobacteria* and family *Succinivibrionaceae* were enriched in cows with extremely low SCC (L_SCC group). The rumen microbiota and rumen fermentations are different in the context of different statuses of udder health, however, this association behind the results needs to be further investigated. The results in this study improve our understanding of the rumen microbiota under different health statuses of the mammary gland.

## Author Contributions

YZ, MX, and JL conceived and designed the study. YZ and MX performed the collection of samples, analyzed the sequencing data, interpreted the data, prepared the figures and tables, and drafted the manuscript. JL helped interpret the data and revise the paper. All authors have read and approved the final manuscript.

### Conflict of Interest Statement

The authors declare that the research was conducted in the absence of any commercial or financial relationships that could be construed as a potential conflict of interest.
