# Supplementary material for: Composition of Rumen Bacterial Community in Dairy Cows With Different Levels of Somatic Cell Counts
Source: Front Microbiol. 2018 Dec 24;9:3217. doi: 10.3389/fmicb.2018.03217 (PMC6312127; doi:10.3389/fmicb.2018.03217)
Supplement: Supplementary file 1 [file Data_Sheet_1.doc]

Supplementary Material

**Composition of rumen bacterial community in dairy cows with different levels of somatic cell counts**

Yifan Zhong^1#^, Mingyuan Xue^1#^, Jianxin Liu^1*^

^1^ Institute of Diary Science, MoE Key Laboratory of Molecular Animal Nutrition, College of Animal Sciences, Zhejiang University, 310058Hangzhou, P. R. China

^#^These authors contributed equally to this work.

**^*^Correspondence:**

Corresponding author:

Dr. Jianxin Liu (Requests for materials should be addressed to: J. X. Liu)

[liujx@zju.edu.cn](mailto:liujx@zju.edu.cn)

**1. Supplementary Figures and Tables**

**1.1 Supplementary Figure**

**Supplementary Figure S1.** Principal coordinates analysis (PCoA) based on unweighted Unifrac distances of OTUs. Samples are indicated by points and colored for different SCC groups. (A) PCoA of the four SCC groups. (B) PCoA of the four SCC groups with SCC1 and SCC4 highlighted. (C) PCoA of L_SCC and H_ SCC groups.

**1.2 Supplementary Tables**

**Supplementary Table S1. Ingredients and nutrient compositions of the experimental diet.**

| Ingredient | % of DM |
| --- | --- |
| Ground corn grain | 24.4 |
| Cottonseed meal | 4.33 |
| Soybean meal | 11.0 |
| Corn silage | 16.7 |
| Alfalfa hay | 13.5 |
| Oat hay | 9.63 |
| Distiller | 8.49 |
| Beet pulp | 8.22 |
| Premix^1^ | 3.79 |
| Chemical Composition, % |  |
| Crude protein | 16.3 |
| Ether extract | 3.31 |
| Neutral detergent fiber | 29.2 |
| Acid detergent fiber | 18.2 |
| Non-fibrous carbohydrate (NFC)^2^ | 43.0 |
| Ca | 0.764 |
| P | 0.521 |
| Net energy for lactation (NE_L_), Mcal/kg DM | 1.70 |

^1^ Formulated to provide (per kilogram of DM) Fe 600 mg; Cu 650 mg; Mn 630 mg; Zn 3,000 mg; Se 17 mg; I 36 mg; Co 8 mg; Vitamin A 250,000 IU; Vitamin D 50,000 IU; Vitamin E 1,100 IU; 15~18% NaCl; water <10%.

^2^ NFC=100 - % NDF - % CP - % ether extract - % ash.

**Supplementary Table S2.** **Power analysis of the outcome variables when comparing L_SCC and H_SCC groups.**

| Variables | Total sample size | Power |
| --- | --- | --- |
| Alpha diversity |  |  |
| Shannon | 40 | 0.684 |
| Simpson | 40 | 0.081 |
| Ace | 40 | 0.301 |
| Chao1 | 40 | 0.295 |
| Top 5 phylum |  |  |
| *Proteobacteria* | 40 | 0.78 |
| *Firmicutes* | 40 | 0.158 |
| *Bacteroidetes* | 40 | 0.455 |
| *Tenericutes* | 40 | 0.748 |
| *Spirochaetes* | 40 | 0.104 |
| Top 10 genus |  |  |
| *Prevotella* | 40 | 0.231 |
| norank_o__*Clostridiales* | 40 | 0.095 |
| *Ruminococcus* | 40 | 0.303 |
| norank_f__*Succinivibrionaceae* | 40 | 0.753 |
| norank_f__*Ruminococcaceae* | 40 | 0.372 |
| norank_o__*Bacteroidales* | 40 | 0.261 |
| norank_f__*Lachnospiraceae* | 40 | 0.344 |
| unclassified_o__*Clostridiales* | 40 | 0.799 |
| *Butyrivibrio* | 40 | 0.191 |
| unclassified_o__*Bacteroidales* | 40 | 0.623 |
| VFAs concentrations |  |  |
| Acetate | 40 | 0.114 |
| Propionate | 40 | 0.692 |
| Isobutyrate | 40 | 0.076 |
| Butyrate | 40 | 0.144 |
| Isovalerate | 40 | 0.151 |
| Valerate | 40 | 0.701 |
| Total VFA | 40 | 0.255 |
| A: P ratio | 40 | 0.803 |

**Supplementary Table S3. Unique bacterial genera in 4 groups with different levels of somatic cell counts (SCC).**

| Genus^1^ | Relative abundance (%) |
| --- | --- |
| SCC1 |  |
| unclassified_c__TM7-1 | 0.000186 |
| unclassified_o__SM2F09 | 0.000116 |
| *Flavobacterium* | 0.000116 |
| unclassified_o__*Acidimicrobiales* | 0.000093 |
| *Trichococcus* | 0.000093 |
| unclassified_f__OM27 | 0.000093 |
| unclassified_f__*Beijerinckiaceae* | 0.000093 |
| unclassified_c__TM7-3 | 0.000093 |
| unclassified_f__*Peptococcaceae* | 0.000070 |
| *Gluconobacter* | 0.000070 |
| unclassified_f__*Cryomorphaceae* | 0.000047 |
| *Microvirgula* | 0.000047 |
| unclassified_o__*Chromatiales* | 0.000047 |
| unclassified_f__[*Chthoniobacteraceae*] | 0.000047 |
| unclassified_o__Sva0725 | 0.000023 |
| unclassified_o__*Solirubrobacterales* | 0.000023 |
| unclassified_f__*Micromonosporaceae* | 0.000023 |
| unclassified_f__*Dermatophilaceae* | 0.000023 |
| unclassified_f__*Porphyromonadaceae* | 0.000023 |
| unclassified_o__*Chloroflexales* | 0.000023 |
| *Gracilibacter* | 0.000023 |
| *Ammoniphilus* | 0.000023 |
| unclassified_f__[*Chromatiaceae*] | 0.000023 |
| unclassified_f__*Legionellaceae* | 0.000023 |
| Proteus | 0.000023 |
| unclassified_f__*Rhodocyclaceae* | 0.000023 |
| unclassified_f__*Methylophilaceae* | 0.000023 |
| unclassified_o__*Thiohalorhabdales* | 0.000023 |
| unclassified_c__TM7-3 | 0.000023 |
| SCC2 |  |
| *Ralstonia* | 0.000166 |
| *Sphingobium* | 0.000083 |
| *Lactococcus* | 0.000083 |
| *Rheinheimera* | 0.000083 |
| *Bilophila* | 0.000083 |
| unclassified_f__*Flavobacteriaceae* | 0.000083 |
| SCC3 |  |
| *Helcococcus* | 0.000166 |
| unclassified_c__SC3 | 0.000166 |
| unclassified_c__ABY1 | 0.000166 |
| *Magnetospirillum* | 0.000166 |
| *Prauseria* | 0.000083 |
| *Desulfococcus* | 0.000083 |
| *Anaerotruncus* | 0.000083 |
| [*Prevotella*] | 0.000083 |
| unclassified_o__[*Roseiflexales*] | 0.000083 |
| *Mycoplasma* | 0.000083 |
| SCC4 |  |
| unclassified_f__R4-45B | 0.000089 |
| *Paludibacter* | 0.000089 |
| *Parvibaculum* | 0.000089 |
| unclassified_c__OPB41 | 0.000089 |
| unclassified_o__MBA08 | 0.000089 |
| unclassified_f__*Cytophagaceae* | 0.000089 |
| *Candidatus*_*Koribacter* | 0.000089 |
| *Brevibacterium* | 0.000089 |

^1^ SCC1: somatic cell counts< 200,000/mL; SCC2: somatic cell counts range from 200,001 to 500,000/mL; SCC3: somatic cell counts range from 500,001 to 1,000,000/mL; SCC4: somatic cell counts >1,000,000/mL

**Supplementary Table S4. Unique bacterial genera in cows with the highest (L_SCC) and lowest somatic cell counts (H_SCC).**

| Genus | Relative abundance (%) |
| --- | --- |
| L_SCC |  |
| unclassified_f__LD19 | 0.002038 |
| *norank*_f__*Oxalobacteraceae* | 0.001631 |
| *Enterococcus* | 0.001224 |
| *Rhodobacter* | 0.001020 |
| unclassified_o__*Burkholderiales* | 0.001019 |
| unclassified_o__*Actinomycetales* | 0.000816 |
| unclassified_f__*Lactobacillaceae* | 0.000612 |
| *Fusobacterium* | 0.000612 |
| *Methylosinus* | 0.000612 |
| *Porphyromonas* | 0.000611 |
| *Candidatus_Aquiluna* | 0.000408 |
| *Saccharopolyspora* | 0.000408 |
| unclassified_f__*Cryomorphaceae* | 0.000408 |
| unclassified_o__*Chlorophyta* | 0.000408 |
| *Oribacterium* | 0.000408 |
| *Veillonella* | 0.000408 |
| *Methylobacterium* | 0.000408 |
| *Rhodoferax* | 0.000408 |
| *Polynucleobacter* | 0.000408 |
| *Actinomyces* | 0.000408 |
| *Granulicatella* | 0.000408 |
| *Phascolarctobacterium* | 0.000408 |
| unclassified_o__*Acidimicrobiales* | 0.000204 |
| *Brachybacterium* | 0.000204 |
| *Curtobacterium* | 0.000204 |
| *Arthrobacter* | 0.000204 |
| unclassified_o__*Gaiellales* | 0.000204 |
| unclassified_f__*Chitinophagaceae* | 0.000204 |
| unclassified_f__*Saprospiraceae* | 0.000204 |
| unclassified_f__[*Barnesiellaceae*] | 0.000204 |
| *Dysgonomonas* | 0.000204 |
| unclassified_f__*Porphyromonadaceae* | 0.000204 |
| *Flavobacterium* | 0.000204 |
| unclassified_f__*Flavobacteriaceae* | 0.000204 |
| unclassified_o__*Sphingobacteriales* | 0.000204 |
| unclassified_f__*Chlamydomonadaceae* | 0.000204 |
| *Exiguobacterium* | 0.000204 |
| unclassified_o__*Bacillales* | 0.000204 |
| *Planomicrobium* | 0.000204 |
| *Carnobacterium* | 0.000204 |
| *Trichococcus* | 0.000204 |
| *Gracilibacter* | 0.000204 |
| unclassified_f__*Peptococcaceae* | 0.000204 |
| *Thalassospira* | 0.000204 |
| unclassified_f__*Beijerinckiaceae* | 0.000204 |
| *Rhodovulum* | 0.000204 |
| *Gluconobacter* | 0.000204 |
| unclassified_f__*Acetobacteraceae* | 0.000204 |
| *Azospirillum* | 0.000204 |
| *Schlegelella* | 0.000204 |
| unclassified_f__*Methylophilaceae* | 0.000204 |
| *Microvirgula* | 0.000204 |
| C39 | 0.000204 |
| unclassified_f__OM27 | 0.000204 |
| unclassified_f__[*Chromatiaceae*] | 0.000204 |
| unclassified_o__*Chromatiales* | 0.000204 |
| *Serratia* | 0.000204 |
| *Halomonas* | 0.000204 |
| unclassified_f__*Pasteurellaceae* | 0.000204 |
| unclassified_f__*Pseudomonadaceae* | 0.000204 |
| unclassified_c__*Spirochaetes* | 0.000204 |
| unclassified_f__*Synergistaceae* | 0.000204 |
| unclassified_c__TM7-1 | 0.000204 |
| unclassified_c__TM7-3 | 0.000204 |
| H_SCC |  |
| unclassified_f__*Pirellulaceae* | 0.001020 |
| *Planktothrix* | 0.000612 |
| *Sphingomonas* | 0.000612 |
| *Pediococcus* | 0.000408 |
| unclassified_o__*Rhizobiales* | 0.000408 |
| unclassified_c__*Verruco*-5 | 0.000408 |
| *Collinsella* | 0.000408 |
| *Thermus* | 0.000204 |
| unclassified_f__*Koribacteraceae* | 0.000204 |
| *Cellulomonas* | 0.000204 |
| unclassified_f__*Microbacteriaceae* | 0.000204 |
| *Mycobacterium* | 0.000204 |
| *Sanguibacter* | 0.000204 |
| *Streptomyces* | 0.000204 |
| *Slackia* | 0.000204 |
| unclassified_c__SJA-176 | 0.000204 |
| unclassified_p__*Armatimonadetes* | 0.000204 |
| *Paludibacter* | 0.000204 |
| unclassified_f__*Porphyromonadaceae* | 0.000204 |
| *Wautersiella* | 0.000204 |
| *Sphingobacterium* | 0.000204 |
| unclassified_o__*Bacillales* | 0.000204 |
| *Facklamia* | 0.000204 |
| unclassified_f__*Lactobacillaceae* | 0.000204 |
| unclassified_p__*Firmicutes* | 0.000204 |
| unclassified_f__R4-45B | 0.000204 |
| unclassified_o__*Desulfuromonadales* | 0.000204 |
| HTCC | 0.000204 |
| unclassified_f__*Enterobacteriaceae* | 0.000204 |
| unclassified_c__*Gammaproteobacteria* | 0.000204 |
| unclassified_f__R4-41B | 0.000204 |
